# Combining contour and region for closed boundary extraction of a shape

**DOI:** 10.3389/fpsyg.2023.1198691

**Published:** 2023-11-15

**Authors:** Doreen Hii, Zygmunt Pizlo

**Affiliations:** Visual Perception Laboratory, Department of Cognitive Sciences, University of California, Irvine, Irvine, CA, United States

**Keywords:** boundary extraction, contour, color, log-polar representation, Dijkstra algorithm

## Abstract

This study explored human ability to extract closed boundary of a target shape in the presence of noise using spatially global operations. Specifically, we investigated the contributions of contour-based processing using line edges and region-based processing using color, as well as their interaction. Performance of the subjects was reliable when the fixation was inside the shape, and it was much less reliable when the fixation was outside. With fixation inside the shape, performance was higher when both contour and color information were present compared to when only one of them was present. We propose a biologically-inspired model to emulate human boundary extraction. The model solves the shortest (least-cost) path in the log-polar representation, a representation which is a good approximation to the mapping from the retina to the visual cortex. Boundary extraction was framed as a global optimization problem with the costs of connections calculated using four features: distance of interpolation, turning angle, color similarity and color contrast. This model was tested on some of the conditions that were used in the psychophysical experiment and its performance was similar to the performance of subjects.

## 1 Introduction

**Figure 1:**
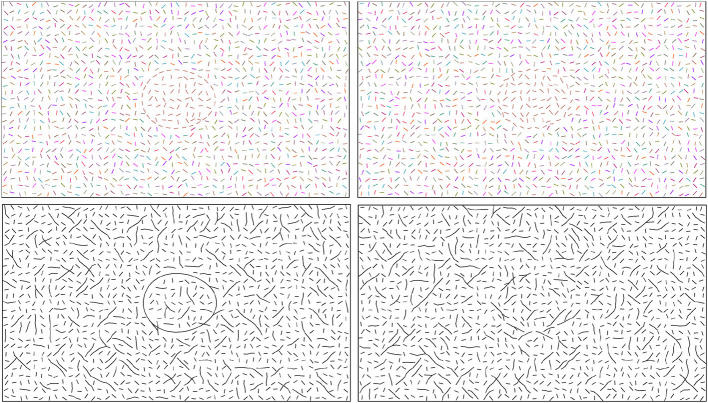
**Top row**: Examples of stimuli used in our experiment. Contrast was reversed in the actual experiment. **Top left**: Orientation jitter of the edges in the boundary of the shape is zero. **Top right**: The orientation jitter is 20°. **Bottom row**: Outputs of a local interpolation algorithm that connected neighboring edges when turning angle was ≤40°. **Bottom left**: This local interpolation was able to extract the boundary of the target egg. **Bottom right**: Local interpolation failed to extract the boundary of the egg, demonstrating the need for global operations in extracting the boundaries in our experiments.

Boundary extraction involves identifying and connecting a set of visual elements such as line edges to form the boundary of an object. Boundary extraction is one of the first, if not the very first operations that the human visual system performs. Given the vast amount of information present in any visual scene, the computations performed by the human visual system must be robust. Specifically, the visual system must be able to ignore irrelevant information and it should be insensitive to errors which could occur during edge detection. Top row in Figure 1 illustrates how our stimuli looked. The egg-like shape in the left panel is easier to see than the one in the right panel. This is because the orientations of the edges defining the boundary of the shape were perfect in the left panel while the orientations were randomly perturbed in the right panel. When orientations of edges form a smooth contour, local interpolation could extract the target boundary (Bottom left of Figure 1). Local interpolation would fail when jitter level is high (Figure 1 Bottom right), highlighting the need for global operations in extracting the boundary. This study investigated human global operations in boundary extraction with a focus on the integration of contour and region information.

In general, the human visual system may use two types of information to accomplish boundary extraction: contour information such as that encoded in edges and region information such as that encoded in color (Grossberg and Mingolla, 1985). Since humans are able to extract boundaries in isochromatic and isoluminant stimuli, the visual system can use either type of information to independently arrive at a boundary solution. Nonetheless, since both contour and color are available in most cases of everyday life, they are jointly encoded in all areas of the lower (V1-V4) and higher regions (lateral and ventral occipitotemporal) of the ventral visual stream (Taylor and Xu, 2022).

Perceptually, when both contour and color are available, the effect of contours seems to dominate over that of color. The McCullough Effect is such an example where the afterimage after viewing two regions with different line orientations and colors depends on the orientation of the lines (Tyler and Solomon, 2019). Further evidence was provided by Vergeer et al. (2015) who demonstrated malleable color percept: different placements of edges created different color percepts, and color inside the shape boundary was always consistent. In fact, previous research has suggested that shape from contour is the fastest cue available to the visual system (Elder, 2018) and is the necessary prerequisite before color-based processing (Moutoussis, 2015). While contour information may adequately suggest a boundary in many cases, color may improve performance when edges are noisy. Hansen and Gegenfurtner (2009) have shown that the two pieces of information are not redundant copies of each other. For example, color is less sensitive to changes in shading or lighting. Therefore, changes in color better indicate a change in material which could in turn suggest the presence a new object (Moutoussis, 2015). Moreover, Taylor and Xu (2023) have found that the cortical areas in the ventral visual pathway could increase the relative coding strength for color depending on the type of stimuli being presented (simple orientation or complex tessellation patterns).

In this study, we (1) performed psychophysical experiments investigating the integration of contour and color using conditions where spatially global operations are required, as well as (2) developed a computational model to emulate human performance. Results from our psychophysical experiments showed that while contour and color information could be utilized in isolation, performance was highest and most robust when they worked in conjunction. Moreover, our results also suggested that contour and color could be integrated only with foveal viewing. With peripheral viewing, performance dropped from ceiling to chance when the orientation jitter increased from 0° to 20°. The failure of boundary discrimination with peripheral viewing could not be explained by a decrease in visual resolution.

Following the study by Kwon et al. (2016), our model uses the log-polar representation of the retinal image. It is known that a log-polar map is a good approximation of the transformation from the retinal image to the early visual areas in the cortex (starting with V1), the first areas responsible for extraction of contours and boundaries (Schwartz, 1977). The log-polar transformation preserves spatially local relations, which means that local neighborhoods in the retinal image are mapped into local neighborhoods in the visual cortex. It follows that there may only be small differences in how computational models work when using the retinal versus the log-polar representation. However, spatially global computations may look very different in the retinal (Cartesian) coordinate system versus in the cortical (log-polar) coordinate system because the log-polar mapping distorts spatially global relations^1^. Not only so, we believe that the concept of log-polar is tightly related to other known visual architectures such as the multiresolution / multiscale pyramid (Rosenfeld and Thurston, 1971; Tanimoto and Pavlidis, 1975).^2^ In the present study we emphasized *spatially global computations that result in a closed boundary of a 2D region on the retina*. We further argue that the log-polar representation is essential in guaranteeing a closed boundary solution when spatially local computations are insufficient (such as that in Figure 1 right column).

We substantially elaborated the previous model proposed by Kwon et al. (2016). Similar to Kwon et al. (2016), the current study focused on the conditions which are perceptually difficult, namely when orientation jitter was added to remove local contour cues. Therefore, the target shape in our stimuli would be difficult or even impossible to detect for a computational model that uses only local operations (see Figure 1). Additionally, we showed that subjects' performance improved when color information was made available. Thus, two requirements were placed on the computational model: (1) the model must perform global operations to be able to accurately produce a closed boundary, and (2) the model must be able to combine both contour and color information. Our proposed model guarantees closure and implements five other Gestalt principles including proximity, good continuation, convexity, color similarity and dissimilarity. This model was tested on some of the conditions on which the subjects were tested, and its performance was similar to the performance of the subjects. We additionally tested the model on a small set of real images and demonstrated promising results. We want to point out that the current model is not intended as the complete theory. Instead, it is the first attempt in capturing the role of contour closure, proximity, good continuation, convexity, color similarity and dissimilarity in the log-polar representation.

## 2 Psychophysical experiment

We extended the experiments reported in Kwon et al. (2016) where the authors measured the role of contour in boundary extraction using black-and-white line drawings. We first replicated their main result, and then performed a 2 × 2 factorial experiment involving two levels of orientation jitter applied to edges and two levels of background colors. We expected that the addition of color information would improve the performance of boundary extraction.

We followed the procedure described in Experiment 3 of Kwon et al. (2016) to test boundary extraction using the fragmented boundary of an egg-like shape embedded in noise edges. The subject's task was to indicate if the pointy side of the egg was oriented to the left or to the right. This task required extraction of the entire boundary of the shape.

### 2.1 Methods

#### 2.1.1 Stimuli

The stimulus consisted of boundary edges belonging to a target shape embedded in noise. In the current experiment, a stimulus canvas of size of [1,920 × 1,080 pixels] was used. To fill the canvas with noise edges, the canvas was divided into [48 × 27] square grids, each with size [40 × 40 pixels]. A noise edge with random orientation was added to each grid, with the center of the edge coinciding with the center of the grid. The edge was allowed to occupy the central 60% of its grid to prevent coincidental connections of neighboring edges.

The target shape was an egg created by distorting an ellipse (Kozma-Wiebe et al., 2006), using the following formula:


$$\begin{array}{l} \frac{x^{2}}{5^{2}}\times \frac{1}{1\pm kx}+\frac{y^{2}}{4^{2}}=1 \end{array}$$

where *k* is the distortion coefficient such that a larger *k* produces an egg with a more obvious pointy side and makes the discrimination task easier. For three of our four subjects (S1-S3), we used *k* = 0.04, which was the same value used in the previous study (Kwon et al., 2016). Subject S4 was tested with *k* = 0.08. The rectangle circumscribed on the egg was 450 × 360 pixels. The horizontal radius (225 pixels) corresponded to a visual angle of 6.66° when viewed from a distance of 60cm. The continuous, smooth egg boundary was fragmented into straight line segments of similar lengths as the noise edges. Every other egg boundary edge was erased, to produce a support ratio of 0.5. The center of the egg was shifted away from the fixation cross in a random direction, with the maximum shift being 50% of the minor radius of the egg.

Then, orientation noise was added to the edges belonging to the egg boundary. Two levels of orientation jitter were used: 20° and 180°. We followed the convention in Kwon et al. (2016), where an average orientation jitter of 20° referred to random rotation of a boundary edge sampled from either [-25°, -15°] or [+15°, +25°]. Orientation jitter of 20° was chosen because contour smoothness was reported by Kwon et al. (2016) to be ineffective for local interpolation. Therefore, the experiment with this level of jitter would likely measure spatially global operations applied to the entire closed contour at once. Sensitivity to jitter was directly tested in our control experiment (Section 4.3.1). Moreover, local interpolation based on smoothness failed to extract boundary for jitter level of 20° (Figure 1 bottom right). The jitter 180° condition changed the orientation of the edge by an angle between -180° and +180°. This implied that the orientation of the contour fragments of the egg conveyed no information about the boundary of the egg. Once the egg edges were prepared, they were added to the canvas. Noise edges were removed if necessary to prevent overlapping of edges.

At this point, the stimulus canvas consisted of grid-like noise edges and fragmented egg edges. All edges had thickness of one pixel. To better conceal the egg, positional jitter was added to noise edges by moving their centers by a random amount in the range [-10, +10] pixels and in a random direction, with the constraint that no edges overlapped. The resulting stimulus consisted of white edges on a black background. Examples of this stimulus are shown in Figure 3, first row.

##### 2.1.1.1 Adding colors to edges

**Figure 2:**
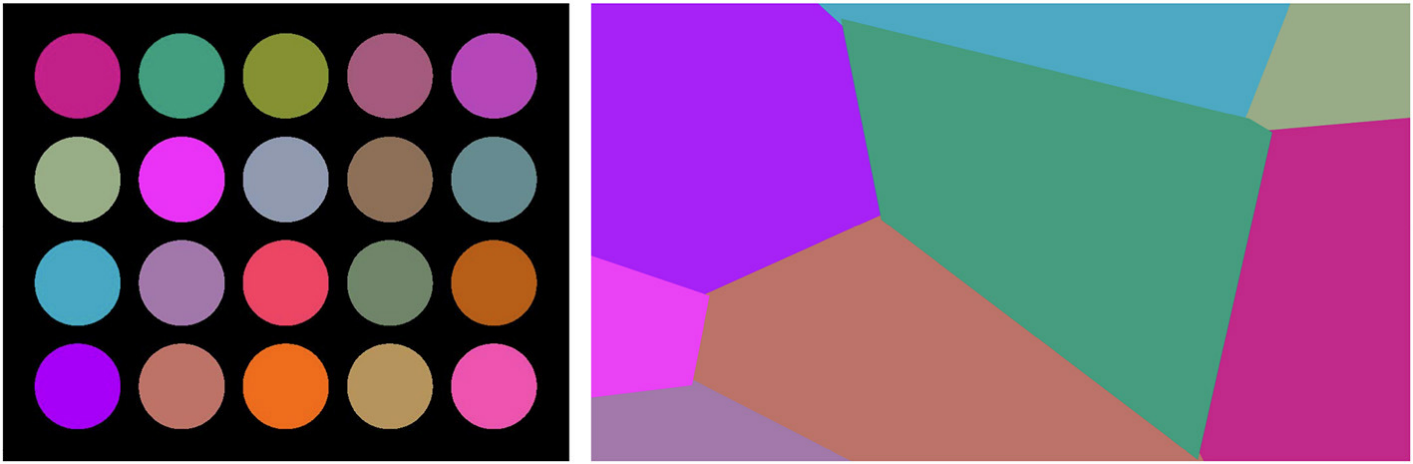
**Left**: Custom colormap of 20 colors used in this experiment. **Right**: An example of a Worley color pattern created by coloring eight Voronoi partitions.

The edges inside the egg (including boundary edges) were colored as follows. In each trial, a new, randomly generated Worley noise pattern was used. Worley noise is a popular texture generation method to simulate real world patterns (Worley, 1996). In this study, we generated Worley color pattern by performing Voronoi partition using a set of five to nine seed points randomly positioned on the canvas. Each Voronoi partition was given a different color to generate a Worley color pattern. To make sure that the color regions were clearly visible against a black background and against the white noise edges, we constructed a custom colormap of 20 colors after restricting the categorical colors from Colorcet to only those with brightness values within the range of 40–60 (out of 100) (Bednar et al., 2020). The colors used in our experiments are shown in Figure 2, along with an example of the Worley color pattern. Based on the position of the egg, noise edges inside the egg boundary (including the edges of the boundary) took on the colors as defined by the Worley color pattern. The number of color regions varied depending on the sizes of the Voronoi partitions.

**Figure 3:**
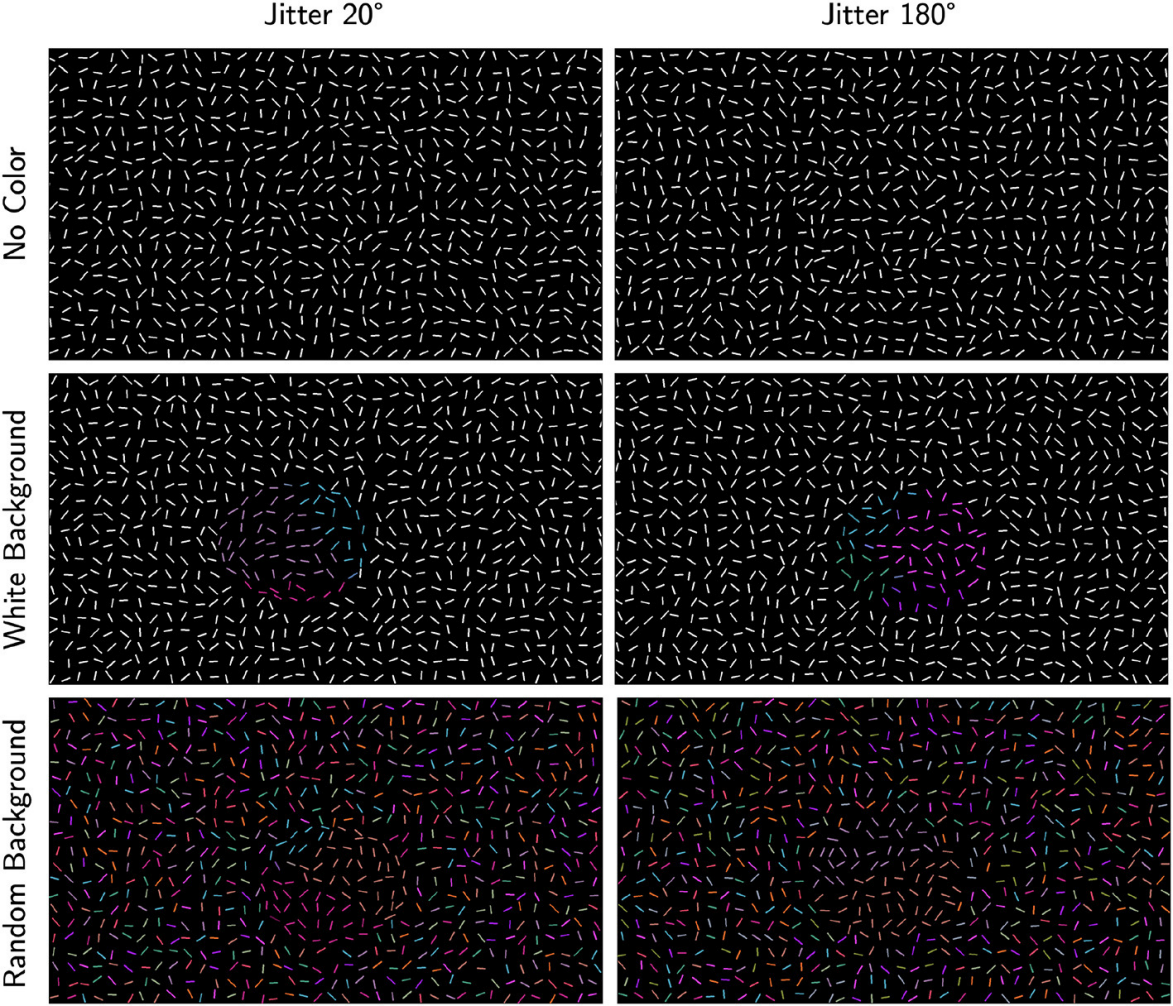
Examples of the stimuli. The **left column** shows examples with jitter 20° and the **right column** shows jitter 180°. The **first row** shows the conditions with no color, the **second** and **third rows** show examples with Worley color pattern added to all edges inside the egg (including the boundary edges). In the **second row**, edges outside the egg are white (white-background) whereas they have random color (random-background) in the **third row**.

We used two color conditions in the background. In one condition, all noise edges in the background were uniformly white (white-background), and in the other condition, each noise edge in the background was assigned a random color from the custom color map (random-background). Examples from both conditions are shown in the second and third rows of Figure 3 respectively. We expected the white-background condition (second row) to be easier than the random-background condition (third row). Note that our stimuli with color looked like watercolor illusions, namely illusory colors spread in between the blank space of edges and filled in the region (Pinna et al., 2001).

We randomized all parameters irrelevant to the manipulated variables. So for each trial, orientation and position jitter of noise edges were sampled randomly; the fragmentation of the egg had a random starting point; each edge belonging to the egg boundary had a random orientation jitter added; a new position for egg center was selected; a new Voronoi partition was generated; random color was sampled to fill each partition when creating a Worley pattern; and if applicable, the colors for noise edges outside the egg were sampled randomly from the colormap.

#### 2.1.2 Experimental conditions

Example stimulus illustrating each of the six experimental conditions is presented in Figure 3. To improve visibility of these examples, edges are drawn with thicker lines and with a lower density of edges relative to the size of the stimulus. So, these images are not copies of our stimuli. Nevertheless, they illustrate the conditions well. The actual stimuli used in the experiment are publicly available. In Figure 3, we show right-pointing eggs in the first and third rows and left-pointing eggs in the second row.

#### 2.1.3 Subjects

Four subjects were tested: Subject S1, who received an extensive practice before data collection; Subject S2; and two naive subjects, Subjects S3 and S4. In the main experiment, three subjects were tested with distortion coefficient *k* = 0.04 and Subject S4 was tested with a larger distortion (distortion coefficient, *k* = 0.08) to make sure that performance in most conditions was well above chance. All subjects had normal or corrected to normal vision.

#### 2.1.4 Procedure

Signal detection experiment was used. Each session consisted of two hundred left-pointing eggs and two hundred right-pointing eggs presented in random order. Each session began with 40 warm-up trials before the 400 experimental trials. The experiments were performed in a well-lit room. Subjects viewed the stimuli with both eyes from a distance of 60cm using a chin-forehead rest. The monitor had a 60Hz refresh rate, and the measured chromaticity coordinates of the RGB primary colors and luminance values for the white point are summarized in Table 1. A trial began by displaying the fixation cross at the center of the monitor. Subjects pressed a key to advance when they were ready. A blank screen was shown for 100ms followed by the stimulus that was shown for 100ms. After that, the blank screen was shown until the subject responded by pressing “Q” if the egg pointed to the left or “P” if it pointed to the right. A beep was sounded after an incorrect response. This sequence was repeated until all 400 trials were completed. Subjects were given as much time as needed to familiarize with the task. Subjects completed one practice session before the actual data collection.

**Table 1:** Chromaticity coordinates of the RGB and luminance values of the monitor.

	**x**	**y**	**Y(*cd*/*m*^2^)**
R	0.64	0.35	59.7
G	0.32	0.60	231
B	0.14	0.06	21.9
W	0.312	0.344	314

Subjects were first tested with jitter 20° and no-color condition (Figure 3 top left) to allow for an estimate of their distortion coefficient, *k*. Subjects S1 and S2 were also tested with the jitter 180° and no-color (Figure 3 top right) to verify that performance in this condition was at chance. The other two subjects (S3 and S4) were not tested in the jitter 180° and no-color condition. After that, each subject completed the four main experimental conditions in random order.

### 2.2 Results

**Figure 4:**
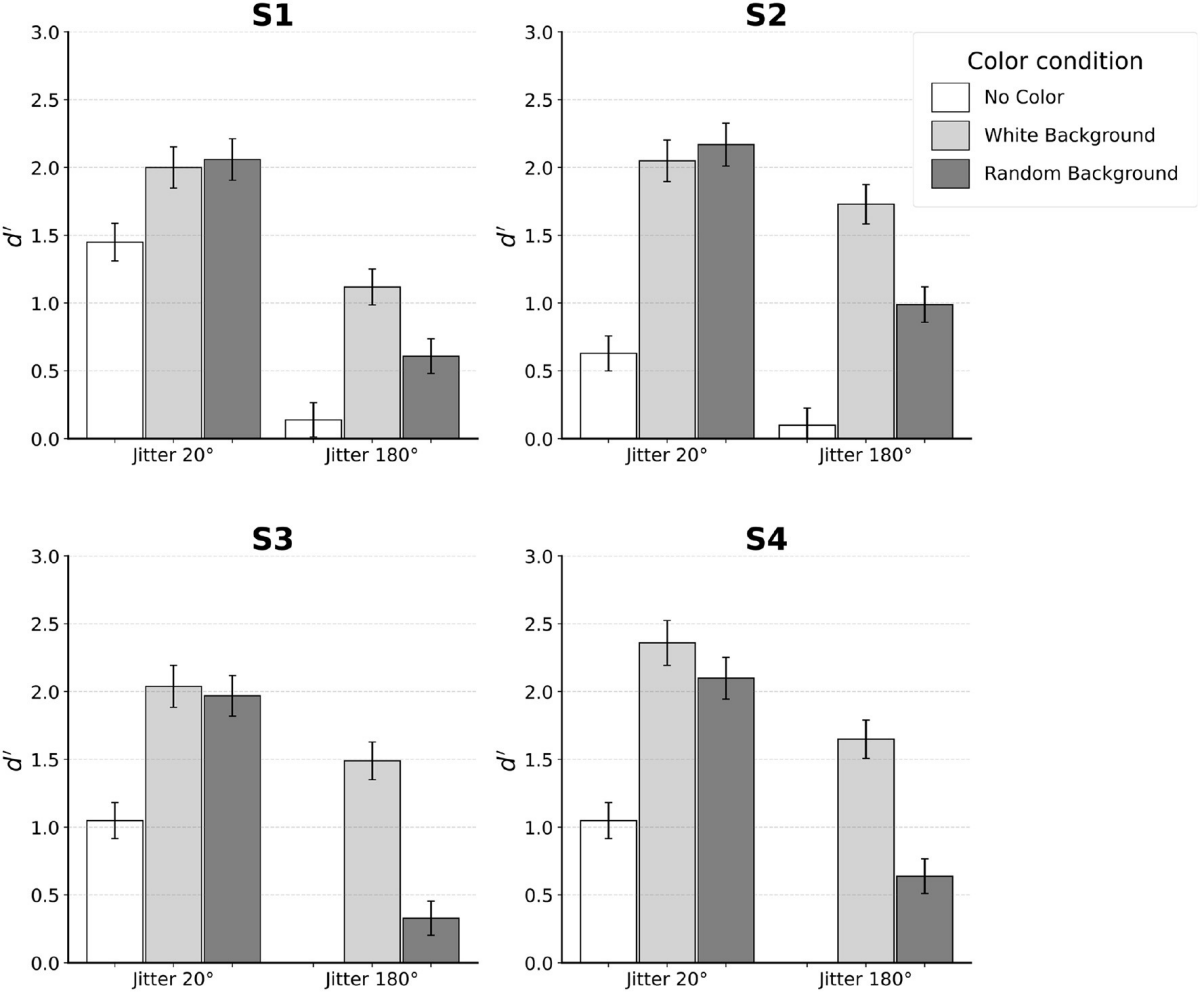
Results for each individual subject. Error bars indicate SE. Subjects S1–S3 performed the experiment with distortion of *k* = 0.04. Subject S4 was tested with *k* = 0.08.

Figure 4 shows the results from individual subjects. Subjects' performance was evaluated using the discriminability measure *d*′ of signal detection. To estimate *d*′ for a two-alternative-forced-choice (2AFC) task, one of the two stimuli (say, egg pointing to the left) can be assigned as “noise” and the other as “signal plus noise”. This way, hit and false alarm rates can be computed and used to estimate *d*′ by subtracting the Z-score of false alarms from the Z-score of hits. A higher *d*′ represents better performance and a *d*′ of zero indicates chance performance. Reliability of *d*′ for each subject was estimated using the standard error of *d*′ as described by Macmillan and Creelman (2004) (p. 325).

When no color was used, performance of the subjects was reliable with jitter 20°. Specifically, all four subjects achieved *d*′ between 0.5 and 1.5 in this condition. In contrast, jitter 180° led to chance performance. This was expected, so only two subjects (S1 and S2) were tested in jitter 180° and no-color condition.

Next, we will describe the four conditions in which the color of edges inside the egg was different from the color of edges outside the egg. For jitter 20°, performance was equally good when the background edges were white and when the background edges had random color. At the same time, performance in these two conditions was clearly better than performance in the no-color condition. In three of the four subjects, this improvement was by a factor of 2 or more. A different pattern of results was observed for jitter 180°. Unlike the chance performance where no color was used, adding color led to performance that was above chance, especially when background was uniformly white. For background with random color, performance was lower by a factor of two on average compared to the condition where background color was white.

### 2.3 Discussion

We replicated the results of Kwon et al. (2016) using the jitter 20° and no-color condition by showing that subjects could reliably perform boundary extraction even with this level of jitter. Jitter 180° and no-color produced chance performance, as expected. This indicated the effectiveness of the noise edges in concealing the egg, so that no confounding cue was available for subjects to complete boundary extraction. Since no color was present in this pair of conditions, we expected performance to rely solely on contour-based processing. While contour-based processing tolerated an orientation jitter of 20°, completely randomizing orientations in the jitter 180° condition made contour integration ineffective. Thus, boundary extraction could not occur. When color information was made available, color-based processing was recruited to improve performance. Since contour-based processing was already recruited in the jitter 20° conditions, adding color reflected the joint operation of contour- and color-based processing. In the jitter 180° conditions, color was the only cue that could lead to contour extraction.

In general, adding color improved performance. However, there was a difference in the magnitude of improvement depending on both the degree of orientation jitter and the background color. Random color in the background was found to modulate performance only when color-based processing operated in isolation (jitter 180°). When both contour- and color-processing operated in conjunction (jitter 20°), performance was equally good in the white-background and the random-background conditions. So, an interaction effect was found: the type of background (white versus random color) had a strong effect for jitter 180°, but not for jitter 20°.

There were individual differences in the way subjects utilized the contour smoothness and color cues. Specifically, Subject S2 relied more on color cue so that his performance with only color-based processing (jitter 180° with both white- and random-backgrounds) was higher than when contour-based processing operated in isolation (jitter 20° and no-color condition). In contrast, Subject S1 relied more on contour smoothness cue so that her performance was higher when contour-based processing operated in isolation than when color-based processing operated in isolation. Subjects S3 and S4 fell between the two extremes: color cue alone led to higher performance than contour alone, only with white background.

Nonetheless, when jitter was 20°, all subjects were able to combine the contour and color information to produce similar level of performance. Individual differences were the smallest when both contour and color information were made available.

## 3 Control experiments

### 3.1 Effect of distortion

**Figure 5:**
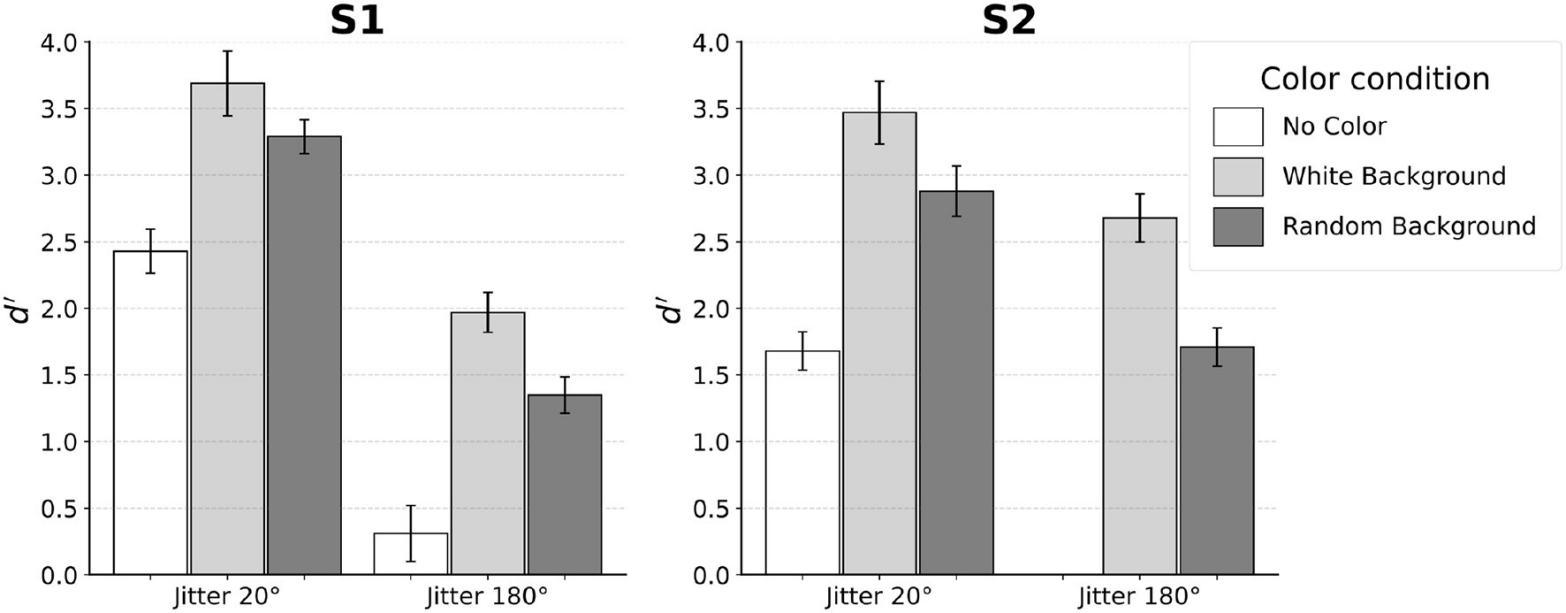
Performance with larger shape distortion (*k* = 0.08). Performance improved, but the pattern of results is the same as with *k* = 0.04.

Two subjects, S1 and S2, who performed the experiment using distortion *k* = 0.04 repeated the experiment with *k* = 0.08. Subject S2 was not tested in jitter 180° no-color condition. The results are shown in Figure 5. Increasing egg distortion made the task easier roughly by a factor of two, but the pattern of results is the same as with *k* = 0.04.

The consistent pattern of results for the two distortion coefficients indicated that the same underlying contour integration mechanism was at play for both distortion levels. Therefore, increasing distortion only improves shape discriminability, making the interpretation of the boundaries easier without altering the processing involved in boundary extraction.

### 3.2 Fixation outside the egg

It was shown that the human visual system could perform local processing based on smooth contours (Field et al., 1993) or color similarity (Kovács, 1996). To determine the role of local versus global processing, we performed a control experiment where fixation was placed outside of the egg. Peripheral viewing precludes the extraction of closed contours using a log-polar representation because the problem can no longer be translated into a shortest path global optimization problem (see Section 4 Model). Therefore, we expected local processing to be a critical mechanism during peripheral viewing.

#### 3.2.1 Methods

**Figure 6:**
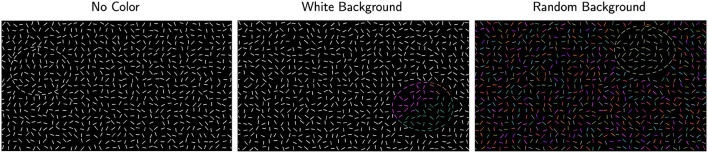
Examples of stimuli with jitter 0° for peripheral viewing. The three color conditions are shown in separate columns: no-color condition where all edges were white, white-background condition where Worley color pattern was added inside the egg (including boundary edges), and random-background condition where Worley color pattern was added inside the egg and edges in the background had random color.

The same stimulus generation procedure was adopted with the exception that now the center of the egg was randomly placed outside a circle covering the central 50% of the stimulus canvas. This way the fixation was always outside the egg boundary. All six experimental conditions were tested. In addition, three experimental sessions with perfectly smooth contour (jitter 0°) were added. Figure 6 illustrates different egg positions using a target egg with jitter 0°. Subject S1 was tested in this experiment.

#### 3.2.2 Results and discussion

**Figure 7:**
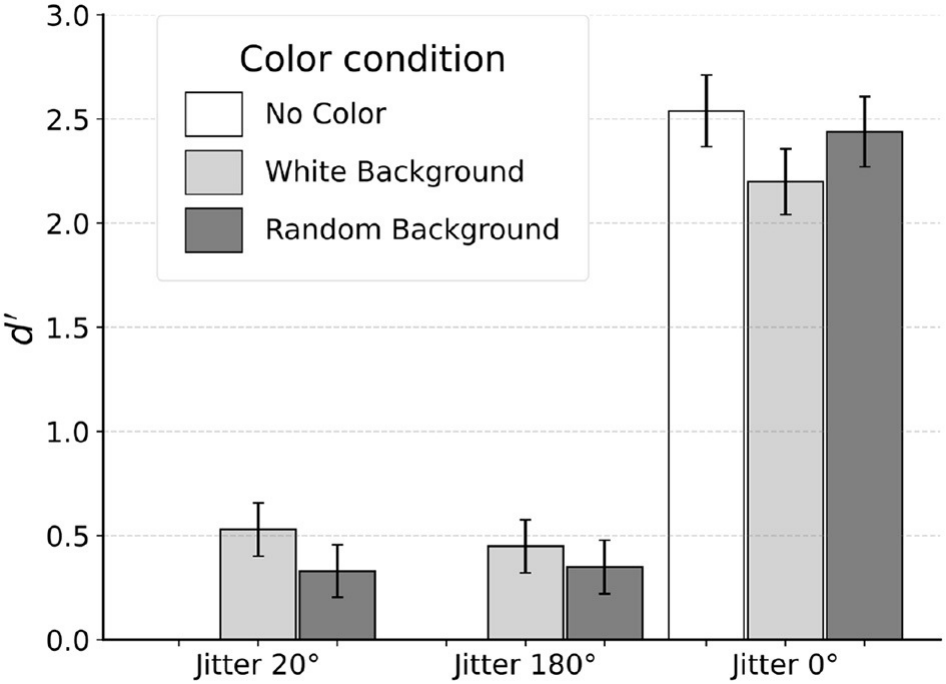
Results from peripheral viewing.

Subject S1 was unable to see the egg in two of the conditions with jitter 20° and jitter 180° that had no color. Thus, no data was actually collected for these conditions. Results from the remaining seven conditions are shown in Figure 7.

For jitter 20°, *d*′ was 0.53 and 0.33 for white-background and random-background conditions respectively. Recall that this subject produced, in the corresponding conditions, *d*′ values of 2.00 and 2.06 when tested with foveal viewing (fixating inside the egg). For jitter 180°, *d*′ was 0.45 and 0.35 for the two color conditions respectively, compared with the *d*′ values of 1.12 and 0.61 with foveal viewing. When tested without orientation jitter (jitter 0°), Subject S1 produced *d*′ values of 2.54, 2.20 and 2.44 for the no-color, white-background and random-background conditions. For comparison, performance was perfect (proportion correct 100%) when jitter 0° was used with foveal viewing.

A decrease in discrimination of checkerboard patterns during peripheral viewing was documented in Schlingensiepen et al. (1986). These authors measured a drop in *d*′ by a factor of two when fixation was outside the stimuli compared to free viewing of the stimuli. In our experiment, fixating outside of the target shape dramatically changed the subject's performance. When tested with jitter 20° and no-color condition, moving the stimulus to the periphery made the stimulus invisible. Adding color helped, only to a small extent. Reliable performance was observed only when smooth contour with jitter 0° was used. We will suggest later in this paper that this change is related to unavailability of the global shortest path optimization when log-polar representation is used. We want to point out that the fact that the egg was invisible in peripheral viewing when there was no color for both jitter 20° and 180° cannot simply be explained by poor visual resolution because the same target shape was clearly visible with jitter 0°.

On top of a decrease in performance, the general pattern of results was different from that with foveal viewing. Specifically, with peripheral viewing, we did not observe an interaction effect between jitter level and the type of background color. These results suggest that the subject had to rely on a completely different mechanism when the fixation was outside the target shape. Specifically, local cues such as smooth contour in the jitter 0° condition had to be used to perform the task. With jitter 20° and greater, local processing based on smoothness is no longer effective. Interestingly, once boundary was smooth, adding color did not improve performance.

We conclude that local processing using contour information could occur in periphery only when sufficiently smooth contours were present. Robustness to orientation jitter could be achieved only when fixation was inside the boundary. Similarly, integration between contour- and color-based processing seemed to occur only when fixation was inside the boundary.

## 4 Model

### 4.1 Model architecture

**Figure 8:**
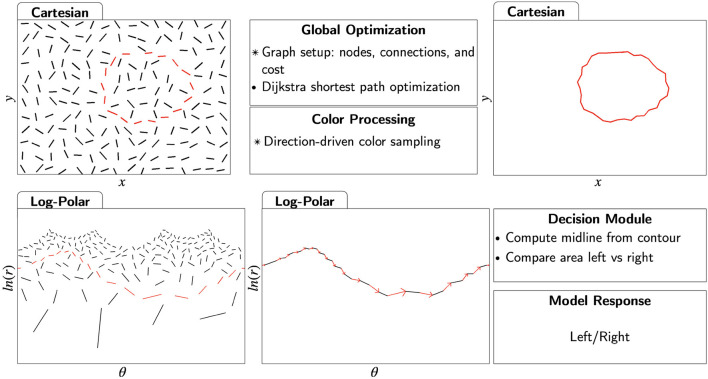
Graphical summary of the model. Operations that were modified in this study compared to the previous study by Kwon et al. (2016) are indicated by asterisks. Edges belonging to the egg boundary are highlighted in red. Given an image, the model receives as input a set of edges and the color values associated with each edge. Then, the model fixates at the center of the stimulus canvas and selects an orientation as its reference angle (0 rad.) to define the log-polar coordinate space. A graph is constructed where a node represents the log-polar coordinates of the two endpoints of an edge along with their associated colors on both sides. Nodes are connected to all other nodes in its neighborhood. Global optimization is then performed using Dijkstra shortest path algorithm to connect the representation of the initial node at reference angle (0 rad.) to its representation at 2*π* rad. The output in Cartesian representation in the form of a closed boundary is obtained by mapping the shortest path solution from log-polar into Cartesian representation. A decision module interprets the boundary output to provide a response if the pointy side of the egg is to the left or to the right.

We extended the biologically-inspired model introduced by Kwon et al. (2016) to include color processing. Similar to the model described by Kwon et al. (2016), our model uses the log-polar representation of the image and solves the least-cost path problem by applying Dijkstra algorithm. The log-polar representation is a good approximation to the retinotopic mapping in the early visual areas of the cortex (Schwartz, 1977). By adopting log-polar representation, the computationally hard problem of extracting a closed boundary is transformed into an easier problem of finding the shortest path. Figure 8 provides a graphical summary of the model implementation. Operations that are different compared to the previous model are labeled with asterisks. We describe the individual steps in the following subsections.

#### 4.1.1 Log-polar

The log-polar representation is a good approximation of the mapping from the retina to the visual cortex (Schwartz, 1977). This is because the linear density of ganglion cells in retina is non-uniform, with hyperbolic decrease (approximately $\frac{1}{r}$) as eccentricity, *r*, increases. Therefore, right at the first step of visual processing is a space-variant sampling of the visual input. This nonuniform sampling is subsequently mapped onto uniformly distributed visual cortical neurons, resulting in an over-representation at the fovea and under-representation at the periphery (cortical magnification). Based on measurements of cortical magnification factors in macaques, Schwartz (1977) demonstrated that the log-polar transformation closely approximates this mapping from the retina to the visual cortex.

The log-polar transformation begins with specifying a polar coordinate system on the image. Instead of using Cartesian coordinates (*x, y*), we use polar coordinates: radius *r* and angle θ. The origin of the *r* dimension represents the center of the retina, which is a projection of the point in the visual field where the eye fixates.

The log-polar coordinate system is defined by taking the logarithm of the *r* dimension. Two requirements must be met for the mapping from Cartesian to log-polar to be the proper transformation as defined in complex analysis: the base of the logarithm must be *e* (i.e., natural logarithm), and the angle θ must be expressed in radians (not degrees). This way, the log-polar mapping is a conformal mapping, preserving local angles. It is precisely this mapping that has been shown to approximate the mapping from the retina to the primary visual cortex. The best way to avoid confusion is to apply a complex-logarithmic function to the complex variable (*z* = *x*+*iy*) representing an image point (*x, y*). Any complex number, *z*, may be expressed in polar form, by using Euler formula: *z* = *x*+*iy* = *r*(cosθ+*i*sinθ) = *re*^*iθ*^. Taking the complex-logarithm of the complex number *z*, using a logarithm to the base of *e* results in *log*_*e*_(*z*) = *log*_*e*_(*r*)+*iθ*. We would like to point out that software packages or libraries often have a function for log-polar transformation, but this function is not necessarily a conformal mapping, namely the logarithm is not natural and/or angle is not expressed in radians.

##### 4.1.1.1 Cartesian to log-polar transformation

The input stimulus with size [1920 × 1080 pixels] was transformed into a log-polar image with size [1,920 × 1,920 pixels]. The model took as input a set of Cartesian coordinates defining the edges detected in the image. In this paper, we used synthetic images described in Section 2.1.1. As a result, our model did not have to perform edge detection because the edges already existed. The model took as input an [*N* × 4] matrix where *N* is the number of edges in the stimulus, and each edge was defined by its two endpoints in the Cartesian coordinates, (*x*_1_, *y*_1_, *x*_2_, *y*_2_).

We then transformed the Cartesian coordinates into log-polar coordinates. We defined *r* = 0 to be the fixation cross, which was placed at the center of the stimulus image. The origin for the polar angle was selected using the same strategy as in the previous study (Kwon et al., 2016). Specifically, θ = 0 was set at the midpoint of a randomly selected starting edge belonging to the boundary of the target egg. This edge was used as the start/end point for computing the shortest path. Kwon et al. (2016) showed that if a starting point was not provided, the model could try a number of starting points and compute the shortest path for all these points. The shortest path from all these paths almost always corresponded to the correct boundary (see their Model LI-SP-EST).

#### 4.1.2 Global optimization

With the new representation in the log-polar space, the original task of boundary extraction was framed as finding the shortest path connecting the representation of the starting edge at 0 rad. back to its representation at 2π rad. Dijkstra shortest path algorithm was used to perform global optimization. Below, we describe the three main components in setting up a graph for optimization: defining the nodes in the graph, establishing connections between nodes, and assigning the costs of travel from one node to another. Connection between nodes is more commonly termed as an “edge" in the context of graph theory. To avoid conflict in terminology, we use the term “edge" when discussing a detected edge in the image; we use the term “connection" to mean the edge from one node to another in a graph.

##### 4.1.2.1 Defining a node in the graph

We defined a node in the graph using three sets of values: an ordered set of endpoints of an edge in the log-polar coordinates [(*r*_1_, θ_1_), (*r*_2_, θ_2_)], and two sets of color values associated with the region to the left and to the right of the edge, RGB (Left), RGB (Right).

To encode contour-related information in the graph, the position and orientation of a detected edge was included in the definition of a node as an ordered set of endpoints in the log-polar coordinates [(*r*_1_, θ_1_), (*r*_2_, θ_2_)]. Since there are two possible directions of travel between two endpoints, each log-polar edge was represented twice in the graph: once for the forward direction, [(*r*_1_, θ_1_), (*r*_2_, θ_2_)], and the other for the reversed direction, [(*r*_2_, θ_2_), (*r*_1_, θ_1_)]. A similar implementation where an edge was represented twice in order to explicitly express direction was described by Williams and Thornber (1999).

**Figure 9:**
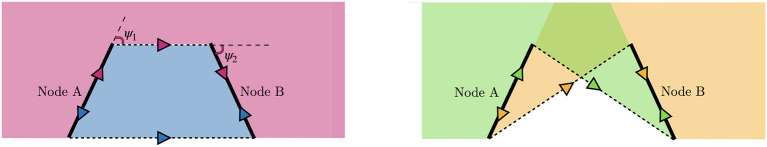
There are four possible permutations representing the interpolation between two edges in the image. Solid line denotes a node in a graph with the direction of travel indicated by the arrow. Dashed lines denote the interpolations. The two angles of interpolation, *ψ*_1_ and *ψ*_2_ are marked. Turning angle is the sum of the absolute values of these angles.

Next, we describe our approach to introduce color-related information in the graph. In particular, we would like to encode color in a way that would preserve the contour-color relationship (Rentzeperis et al., 2014). As an illustration of the contour-color relationship, imagine a white circle placed on a black background. The closed boundary of the circle separates the stimulus canvas into two regions, foreground and background. The region with white color coincides with the area enclosed by the boundary. Therefore, color information does not contradict the contour-defined boundary. In order to distinguish foreground color from background color while respecting contour edges, we propose the notion of directionality, being inspired by Stahl and Wang (2007). Imagine walking on the boundary of a circle clockwise. The white color belonging to the interior region of the circle is always to the right at the walker local frame. Considering the direction of travel allows the two pieces of information from contour and color to be tracked simultaneously: for contour, the orientation of an edge is the unsigned direction; for color, color similarity in the foreground versus background can be tracked by comparing the colors on both sides of an interpolating edge. In Figure 9, the shaded regions indicate the regions to the left of edges according to their respective directions of travel.

For each edge, color was sampled from a Moore neighborhood of range three (7 × 7 grids) in the Cartesian representation (Moore, 1964). Colors to the left and right of the edge were averaged separately to obtain two sets of RGB values.

##### 4.1.2.2 Defining connections in the graph

We restricted the connectivity in the graph, so that a node in the graph can reach only the set of nodes located within its neighborhood. We defined a neighborhood as a square window of 240 × 240 pixels in the log-polar representation. Therefore, instead of constructing a complete graph with connections for every pair of nodes, only nodes that were sufficiently close to each other were connected. Our pilot tests showed that the quality of solutions was not affected, but computation time was greatly improved.

##### 4.1.2.3 Cost of interpolation

To calculate the cost of interpolation (the cost of a connection in the graph) from Node A to Node B, we used the following features: (1) distance, (2) turning angle, (3) color similarity, and (4) color contrast. The value of each feature was multiplied by its weight. We describe the algorithmic computation for each feature, as well as their relationships to the computational level representation of Gestalt principles (Marr, 2010).

Let us begin with contour information encoded in the edges: distance and turning angle. The visual system is more likely to choose a particular interpolation if the distance (length of the interpolation) is short, commonly referred to as the Gestalt principle of proximity (Wertheimer, 1923). We computed the distance as the Euclidean distance from the second endpoint of the first node to the first endpoint of the second node. Since distance is computed after the log-polar transformation, scaling a shape has no effect on the distance metric. This behavior is desirable since proximity principle has been shown to be robust to transformations of scaling (Kubovy et al., 1998). We squared the interpolated distance in the cost function to progressively penalize long interpolations. This produced good results, but the actual shape of this function (polynomial vs. exponential) should be tested in the future.

Turning angle was used in the cost of interpolation because smaller changes in orientation are more likely to be interpolated (Wertheimer, 1923; Elder, 2018). Turning angle was defined as ψ = |ψ_1_|+|ψ_2_|, where ψ_1_ is the angle formed by the first endpoint of Node A, second endpoint of Node A, and first endpoint of Node B; and ψ_2_ is the angle formed by the second endpoint of Node A, first endpoint of Node B, and second endpoint of Node B. The angles ψ_1_, ψ_2_ are also labeled in Figure 9. Minimizing turning angle minimizes abrupt changes in the direction of travel, and thus encodes the Gestalt principle of good continuation.

As a natural consequence of minimizing the turning angle in the log-polar space, Gestalt principle of convexity is encoded implicitly without including additional parameter in the cost function. An easy way to see this is to realize that a circle around the fixation point maps into a straight line in the log-polar representation.

Next, we discuss features related to color-based processing: color similarity and color contrast. Edges are more likely to be connected when they share the same colors on the left side and/or on the right side of the interpolated curve, also described as the Gestalt principle of similarity (Kovács, 1996). The difference in colors between the left side of Node A and left side of Node B were computed as follows: Δ Color(Left) = |RGB(Left)_*A*_−RGB(Left)_*B*_|, and similarly for the right sides of both nodes Δ Color(Right) = |RGB(Right)_*A*_−RGB(Right)_*B*_|. The two differences were combined using a minimum operation, Color similarity = min(Δ Color(Left), Δ Color(Right)). As a result, a pair of edges is considered to share similar color as long as they share similar colors on at least one side.

Finally, edges that carry higher color contrasts between the left and right side are more likely to indicate the presence of a boundary. High contrast relates to the pop-out effect or the Gestalt principle of dissimilarity (Pinna et al., 2022). We compared the colors on both sides of an individual node and computed the color contrast = |RGB(Left)_*A*_−RGB(Right)_*A*_|. We used the negative of color contrast for global minimization. Note that the notion of directionality was not encoded in the computation of color contrast, since the two nodes representing the same edge in both directions have the same value for color contrast.

The total cost for every connection in the graph was calculated by summing the cost across the four features, with weights defined by coefficients. The cost function with their normalizing constants was as follow:


$$\begin{array}{l} a_{1}\left({\text{D}}^{2}/1920\right)+a_{2}\left(\text{TA}/2\pi \right)+a_{3}\left(\text{CS}/255\right)+a_{4}\left(1-\text{CC}/255\right) \end{array}$$

where *a*_1_, *a*_2_, *a*_3_, *a*_4_ are the coefficients of the individual features; D, TA, CS and CC represent the cost of distance, turning angle, color similarity, and color contrast respectively. Since changing the coefficients alters the model behavior, we identify a model by specifying its coefficients. For example, a model ignoring color information would set the coefficients for color similarity and color contrast to zero. For the ease of reporting, we label the model in terms of their coefficients using the following convention [distance, turning angle, color similarity, color contrast]. If the model assigned a coefficient of 1 to both distance and turning angle, it would be labeled as [1,1,0,0].

Note that the magnitudes of the four features in the cost function were very different because of the units used (distance was measured in pixels, angle in radians, and color using 256 digital units). As a result, the values of these features were rescaled by their respective constants to be in comparable ranges. This means that the values of coefficients should not be interpreted literally: e.g. a coefficient value of one for distance and a coefficient value of ten for color similarity does not mean that color similarity is ten times more important than proximity. It is, however, possible to make relative comparisons of the components of the cost function across different conditions: an increase in coefficient for color similarity from a value of two to a value of four (assuming that other coefficients stayed the same) meant that color similarity was twice as important in the second condition than the first.

#### 4.1.3 Producing closed contour as model output

After setting up the graph by defining the nodes, connections, and costs, we applied Dijkstra shortest path algorithm. The algorithm solved a global optimization to produce a least-cost path from a starting node to itself. After the shortest path was transformed into edges in the Cartesian representation, pairs of edges were interpolated using straight line segments to produce a closed boundary. The literature provides more sophisticated interpolation methods that could be used in our model (Sharon et al., 2000; Kimia et al., 2003; Stahl and Wang, 2007; Kalar et al., 2010; Ben-Yosef and Ben-Shahar, 2011; Singh, 2015).

The model guarantees closure because the two endpoints of the least-cost path in log-polar translate to the same point in Cartesian space. Therefore, the boundary extraction solution from the model aligns with the Gestalt principle of closure, such that closed contours are perceptually preferred over open ones (Kovacs and Julesz, 1993).

In summary, by representing the problem in log-polar space and performing global optimization using the proposed cost function, a total of six Gestalt principles were operationalized. They are proximity, good continuation, convexity, color similarity, color dissimilairty, and closure.

#### 4.1.4 Decision module

To produce a response to the 2AFC question, we adopted the same decision criterion used by Kwon et al. (2016). Specifically, the model took the horizontal range of the detected boundary and computed the midpoint. The extracted boundary was divided into two areas by drawing a vertical line. The area of the left half was compared to the area of right half. The pointy side of the egg was the side with a smaller area.

### 4.2 Selecting the parameters of the model

The behavior of the model is determined by four parameters, namely the set of coefficients weighing the four features in the cost function (distance, turning angle, color similarity and color contrast). To examine the effect of each model parameter, we created a separate set of 100 egg stimuli and performed grid search on the experimental conditions. Model performance was estimated using *d*′. Since the main goal of the current study was to explore the integration of contour and region (color), our simulations focused on the conditions with jitter 20°. As explained in Section 2.1.1, jitter 180° completely removed contour information by randomizing the orientations of the edges belonging to the egg boundary. It follows that the subjects had to rely exclusively on the color information. We will analyze this condition in the future.

We first established a baseline for the model performance on the no-color condition using contour-related features (distance and turning angle). We did this for both distortion coefficients: *k* = 0.04 and *k* = 0.08.

**Figure 10:**
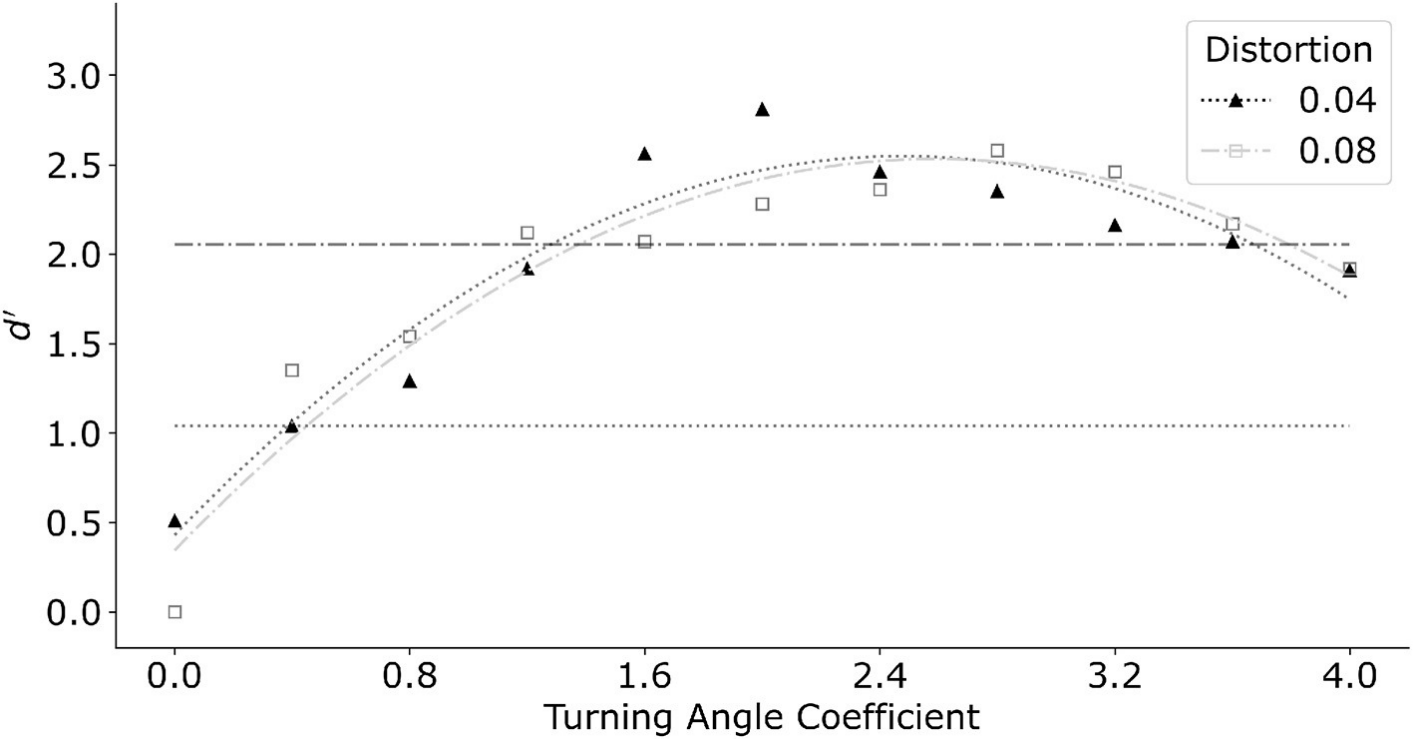
Model performance as a function of increasing turning angle coefficient. Model performance improved initially as turning angle coefficient increased. Further increase of turning angle coefficient degraded performance. Horizontal dotted line and dash-dot line indicate performance of an average subject for distortion *k* = 0.04 and *k* = 0.08 respectively. Turning angle coefficient of 0.4 and 1.2 produced performance closest to that of an average subject, and thus was chosen for subsequent simulations for conditions with color.

Interpolation distance is the dominant feature in the model. The previous version of the log-polar model reported in Kwon et al. (2016) used only the interpolation distance combined with a linear interpolation front-end. Their linear interpolation formed longer contours by connecting approximately collinear edges within a small neighborhood before finding the least-cost path. In the current model, turning angle replaced the linear interpolation. Figure 10 shows the effect of the turning angle coefficient relative to the distance coefficient for two distortions of the egg shape. In this graph we varied the turning angle coefficient from zero to four with a step size of 0.4. Distance coefficient was set to one. Filled triangles represent distortion *k* = 0.04 while open squares represent *k* = 0.08. The same model produced similar performance for both distortion coefficients of the egg. In general, manipulating turning angle coefficient produced a systematic change in performance. The maximum *d*′ was produced at turning angle coefficient of about two. Performance degraded for larger values of the turning angle coefficient. This was related to the model making long interpolations in the log-polar map, producing circular-like parts that did not approximate the egg shape well.

The turning angle coefficients which best captured performance of an average subject was 0.4 for the smaller egg distortion *k* = 0.04, and 1.2 for *k* = 0.08. We therefore fix the turning angle coefficients at the respective values in the subsequent tests which included color. Note that in the main experiment with *k* = 0.04, the three subjects produced *d*′ varying between 0.5 to 1.5. The best performance was produced by S1 who received substantially more practice with these stimuli.

**Figure 11:**
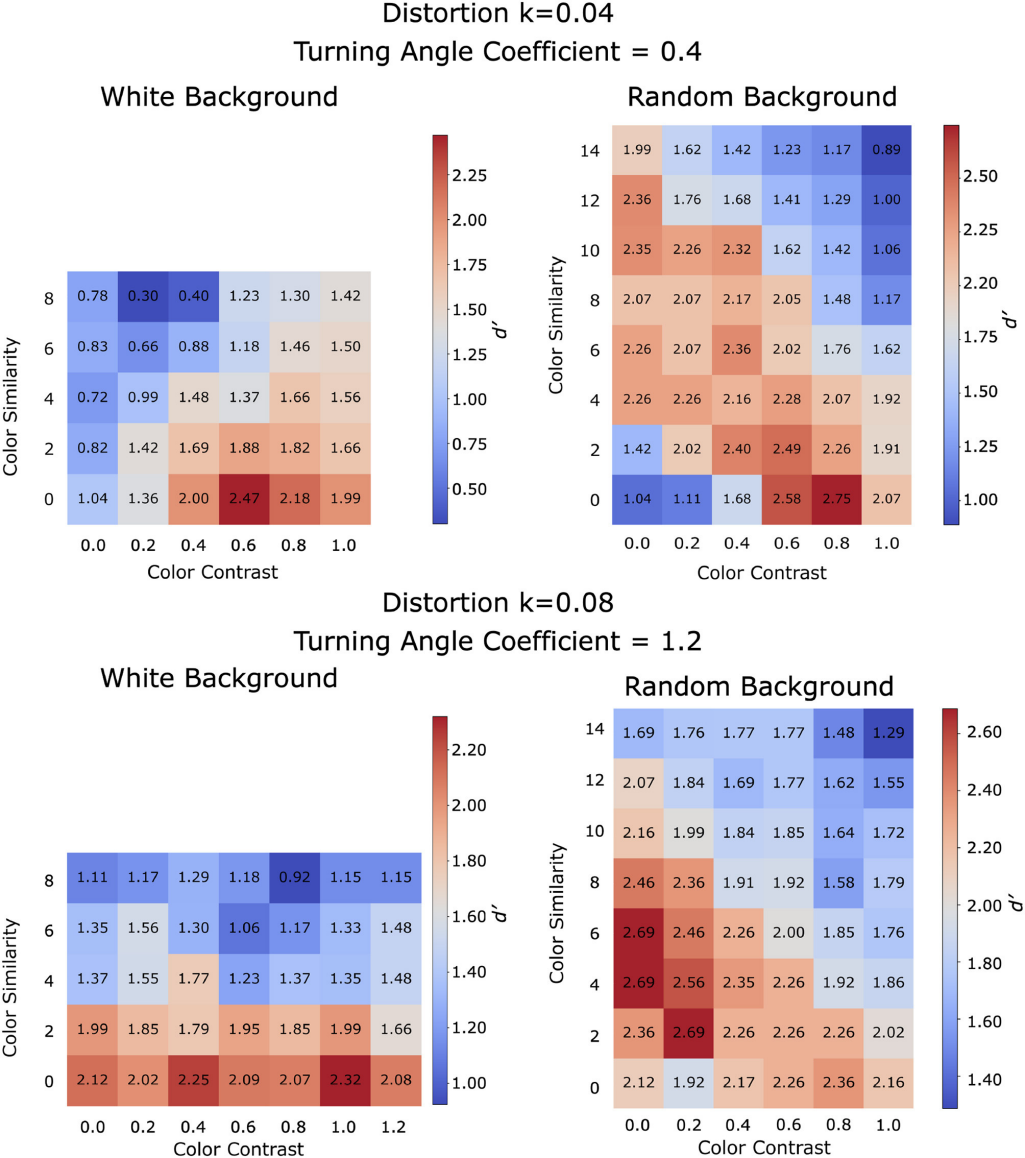
The model parameter space by performing grid search on stimuli with jitter 20°. Model performance was evaluated using *d*′. Results are color coded with red indicating high performance, grey indicating intermediate performance, and blue indicating low performance. **Top**: Grid search results for distortion *k* = 0.04. **Bottom**: Grid search results for distortion *k* = 0.08.

Using the turning angle coefficient identified for each egg distortion (0.4 and 1.2 for distortion *k* = 0.04 and *k* = 0.08 respectively), we performed grid search on color similarity and color contrast coefficients for both white- and random-background conditions (the distance coefficient was set to 1 for all simulations). This grid search was informed by a pilot study exploring a wider range of coefficients. The results for distortion *k* = 0.04 and distortion *k* = 0.08 are summarized in Figure 11. Manipulating color-related coefficients resulted in a gradual change in performance, indicating that the model is stable. For both color conditions, the model was able to combine at least one color feature with contour information to arrive at a higher performance than in the no-color condition. Model performance in the no-color condition is represented by the grid cell where both color similarity and color contrast coefficients are set to zero (bottom left corner of each grid).

**Figure 12:**
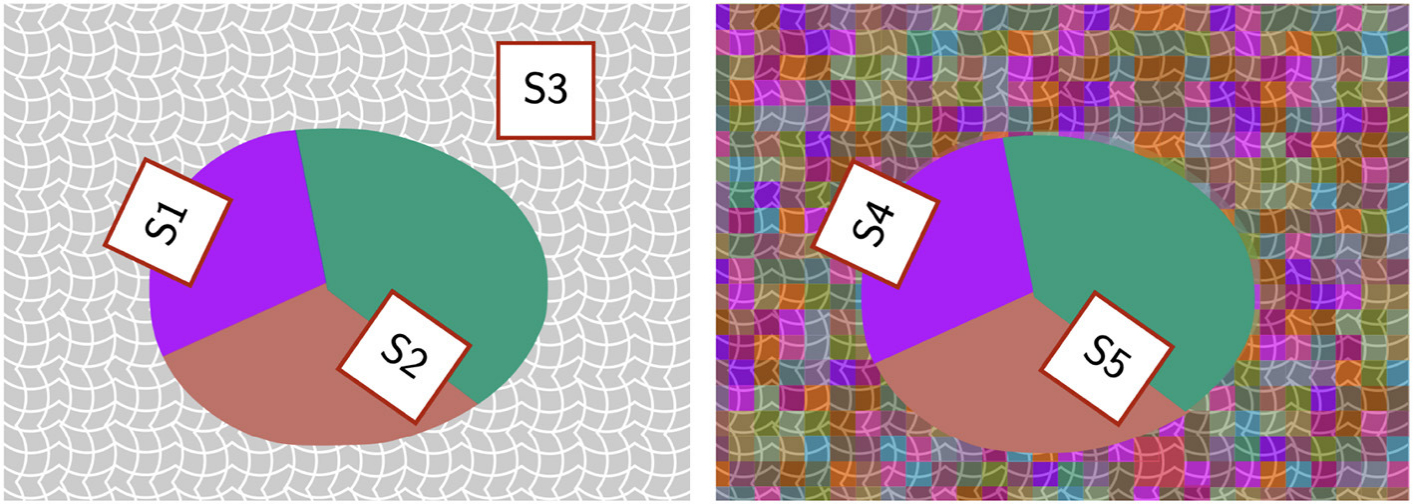
Schematic illustration comparing color similarity and color contrast at different regions of a stimulus. **Left**: White background condition. **Right**: Random background condition. Details described in text.

We will describe the role of color-related coefficients for each color condition separately. For the white-background condition, the model performed well by using positive coefficients for color contrast while ignoring color similarity: increasing color similarity coefficient degraded performance. Since the stimuli consisted of a Worley-colored egg embedded in white background, information about the target shape can be captured well by the color contrast between the inside and outside of the egg (region S1 in Figure 12). Although color contrast could also be high at region S2, the Gestalt principles of convexity and good continuation will bias the solution towards the egg boundary. Increasing the color similarity coefficient (i.e., penalizing color dissimilarity on each side of the contour) also increases the preference to produce a contour passing through the uniformly white noise edges in the background (region S3 in Figure 12). It is important to point out that our white-background condition is computationally simple (see Figure 12, second row), because the model could remove (filter out) all white edges and would be able to extract the shape boundary nearly perfectly with performance close to perfect (the actual performance will not be perfect because the edges of the egg boundary had 20° random orientation jitter). We verified this directly, but the grid search was done without removing white edges in the background.

For the random-background condition, the model can produce high performance for a range of color coefficients. Specifically, increasing color similarity coefficient or increasing color contrast coefficient could both improve performance (Figure 11). This is illustrated by region S4 in Figure 12 where there is color contrast across the boundary and color similarity for the region inside the egg. However, our exploration showed that color-related parameters are limited in their utility. For example, both color similarity and color contrast parameters could bias the solution towards the polygonal shapes inside the egg with Worley color pattern (region S5 in Figure 12) because the polygonal boundaries have high color similarity on both sides and high color contrast across. This could lead to errors in contour integration. Further research is needed to investigate the role of color-related processing in boundary extraction, especially when color introduces geometrical patterns conflicting with the target boundary (e.g., in the case of camouflage).

The results from the parameter space explorations (Figure 11) suggest that the model was able to integrate color with contour features to arrive at a higher performance when compared to contour alone. This improvement was higher with the more difficult case where egg distortion *k* = 0.04. The model coefficients that led to good performance in the grid search were tested in the next section, using the same stimuli which subjects were tested on.

### 4.3 Comparing the model to psychophysical results

Based on the results presented in Figure 11, we applied the model to the images that were shown to subjects for the conditions with jitter 20° with both distortion *k* = 0.04 and 0.08. Because the grid search based on 100 images showed that high performance was achieved with several sets of the coefficients, we applied these sets of coefficients to the 400 images from psychophysical experiment. Differences in model performance across these sets of coefficients were small.

**Figure 13:**
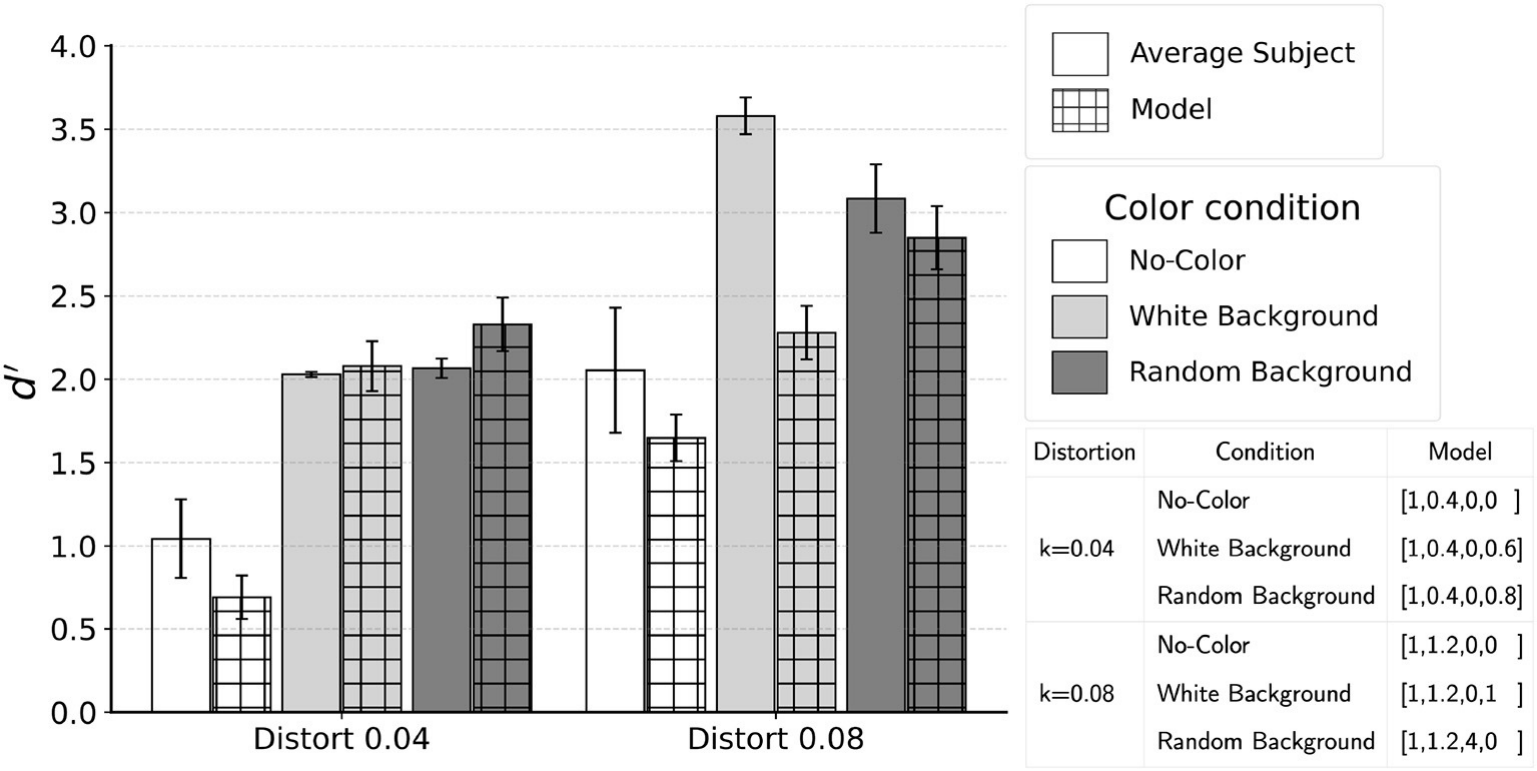
Comparison of the model and subject performance for jitter 20°. Model was able to match performance of an average subject except in one condition with higher distortion *k* = 0.08 and white background. Refer to text for discussion on the interpretation and a plausible model which could perform better for this condition.

In Figure 13, we report the performance for the model coefficients which produced the highest performance in the grid search. The model was successful in matching human performance for five of the six conditions. For distortion *k* = 0.08 white-background condition, subjects performed close to perfect while the model did not. It is possible that the uniform white background noise edges allowed for simple filtering operations to remove the background before extracting contour. We tested the possibility of such pre-processing by applying the model after all white background edges were removed. Using coefficients [1,1.2,0,0], the model produced *d*′ = 3.56, which is almost identical to the average performance of the two subjects who were tested in the control experiment. Future studies can test the hypothesis that the visual system applies a filtering front-end.

To summarize, our results showed that the log-polar based model was successful in integrating contour and color in the test with the egg-like stimuli. The model's performance was not very different from the subjects'. We want to point out that the task was computationally difficult for several reasons: (i) the contour of the egg was fragmented to have a support ratio of 0.5; (ii) the density of the background edges was the same as the density of the edges representing the egg; (iii) the edges representing the contour had random jitter which essentially excluded spatially local growth of the contour based on smoothness; (iv) global optimization was necessary while at the same time avoiding combinatorial explosion related to examining all subsets of edges in the image; (v) the contour had to be closed. Therefore, it is probably not surprising that the model's performance did not exceed that of the best subject. We are confident that our model captured something important about the visual mechanisms of contour integration. However, several components of the model could be further developed and produce even better fit to the subjects' results. To further examine the correlation between the model and the subject, we performed an additional control experiment manipulating jitter level (Section 4.3.1).

#### 4.3.1 Control experiment: effect of jitter

In this control experiment, jitter level was manipulated from 0° to 40° with a step size of 5°. It was natural to expect that increasing jitter (producing non-smooth contours) will lower the performance of subjects. A model which explains how the visual system works would also be affected by jitter level, with a similar degree of quantitative effect.

**Figure 14:**
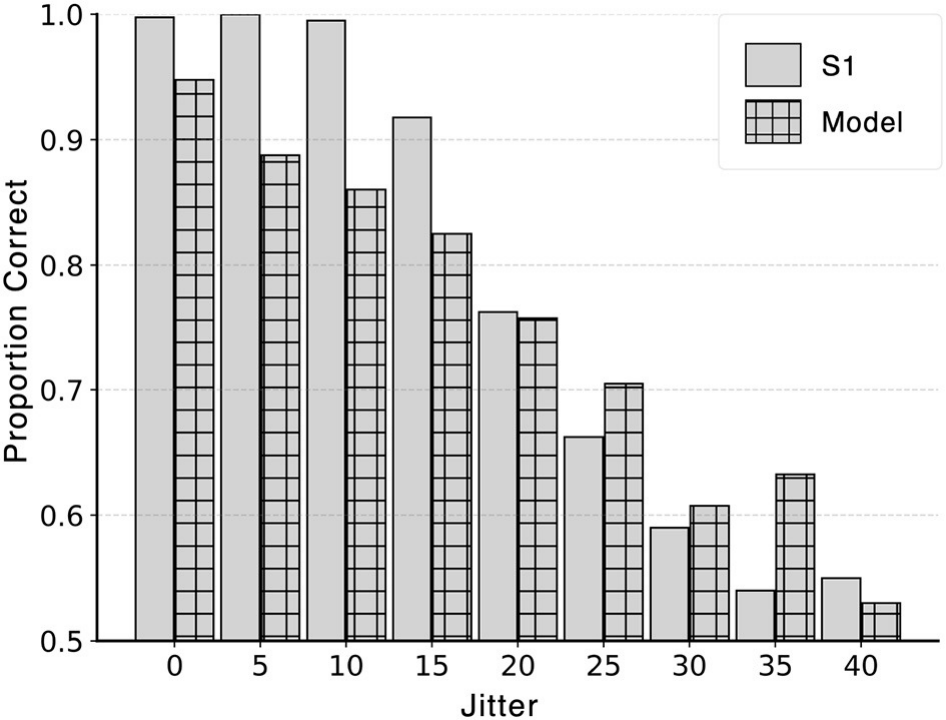
Control experiment investigating the effect of jitter on performance of model and subject. Performance decreased from close to perfect (1.0) to chance (0.5) for both model and subject. Standard error for each bar was no greater than 0.025.

The stimuli for the control experiment were generated using the procedure for distortion *k* = 0.04, No-Color condition described in Section 2.1.1. All target edges in a particular jitter level had a random change in orientation in the range [-jitter - 5°, -jitter + 5°] or [+jitter - 5°, +jitter + 5°] except for jitter level 0°, where no random jitter was added to the target edges. Subject S1 ran additional eight sessions excluding jitter 20°, which was performed as part of the main experiment (Section 2.2). The model with coefficients 1 for distance and 0.8 for turning angle was chosen because the model produced similar performance (*d*′) as subject S1 based on the simulation in Figure 10. The model was tested on all nine conditions and a comparison between model and subject performance is shown in Figure 14.

Performance of both the model and S1 decreased with increasing jitter level. Pearson correlation coefficient between the model and S1's proportion correct was high: R = 0.96 (*p*-value<1E-4). Figure 14 shows overall proportion correct instead of *d*′ because *d*′ approached infinity when S1's performance was close to perfect (either because there was no miss or false alarm) for the first three jitter levels below 15°^3^. This high performance could partially be attributed to the role of local interpolation in boundary extraction. The current model relies exclusively on spatially global optimization. Therefore, adding local interpolation as the front-end would likely produce close to perfect model performance for small jitter levels (see Figure 1). A drop in S1's performance was observed with jitter 15° and above, suggesting that local operations failed with high jitter levels. This result validated the choice of 20° jitter to investigate the role of global processing.

## 5 Conclusion

Given a 2D retinal or camera image, determining which contour and region belong to a single object is the first step to recognizing the object and reconstructing its 3D shape. Our psychophysical experiments eliminated local contour cues by introducing orientation jitter to explore the interaction between edge-based and color-based processing in the context of global processing. We showed that each of these two types of processing could operate in isolation: edge-based processing could reliably extract boundaries when contours were relatively smooth; and color-based processing could reliably extract boundaries when color in the foreground was different than the color in the background. When both contour and color cues were present, subjects were able to integrate the two pieces of information to produce the highest performance. We established these results under viewing conditions where the subject fixated inside the boundary of the object. Moving the fixation outside the boundary substantially impaired subject's performance, but this impairment cannot be explained by a lower visual resolution in the periphery. The design of our experimental stimuli may be extended in future studies. One may manipulate (i) the target 2D shape, (ii) support ratio of the fragmented contour, (iii) the degree of similarity between color inside and outside of the 2D shape, and (iv) the texture pattern for the target shape and the background. These characteristics represent conventional features that have been used to study figure-ground organization.

We proposed a biologically-inspired boundary extraction model combining contour-based processing with color-based processing. The model was tested on the conditions with 20° jitter and its performance was similar to that of the subjects. The main characteristic of the model is the use of the log-polar representation which is known to be a good approximation of the retinotopic mapping in the primary visual areas of the brain. By performing shortest path optimization in the log-polar representation, the model performed global optimization to produce a boundary solution which is guaranteed to close. The model integrated two contour-related features (distance of interpolation and turning angle) and two color-related features (color similarity and color contrast) in its cost function. More specifically, the interaction between contour and color was modeled using the concept of boundary directionality, where the model encoded color as guided by contour-based cues. The model produced reliable results comparable to that of subjects with the two difficult conditions of no color and random background when jitter was 20°. We hope that these results will stimulate further explorations of competing boundary extraction models.

**Figure 15:**
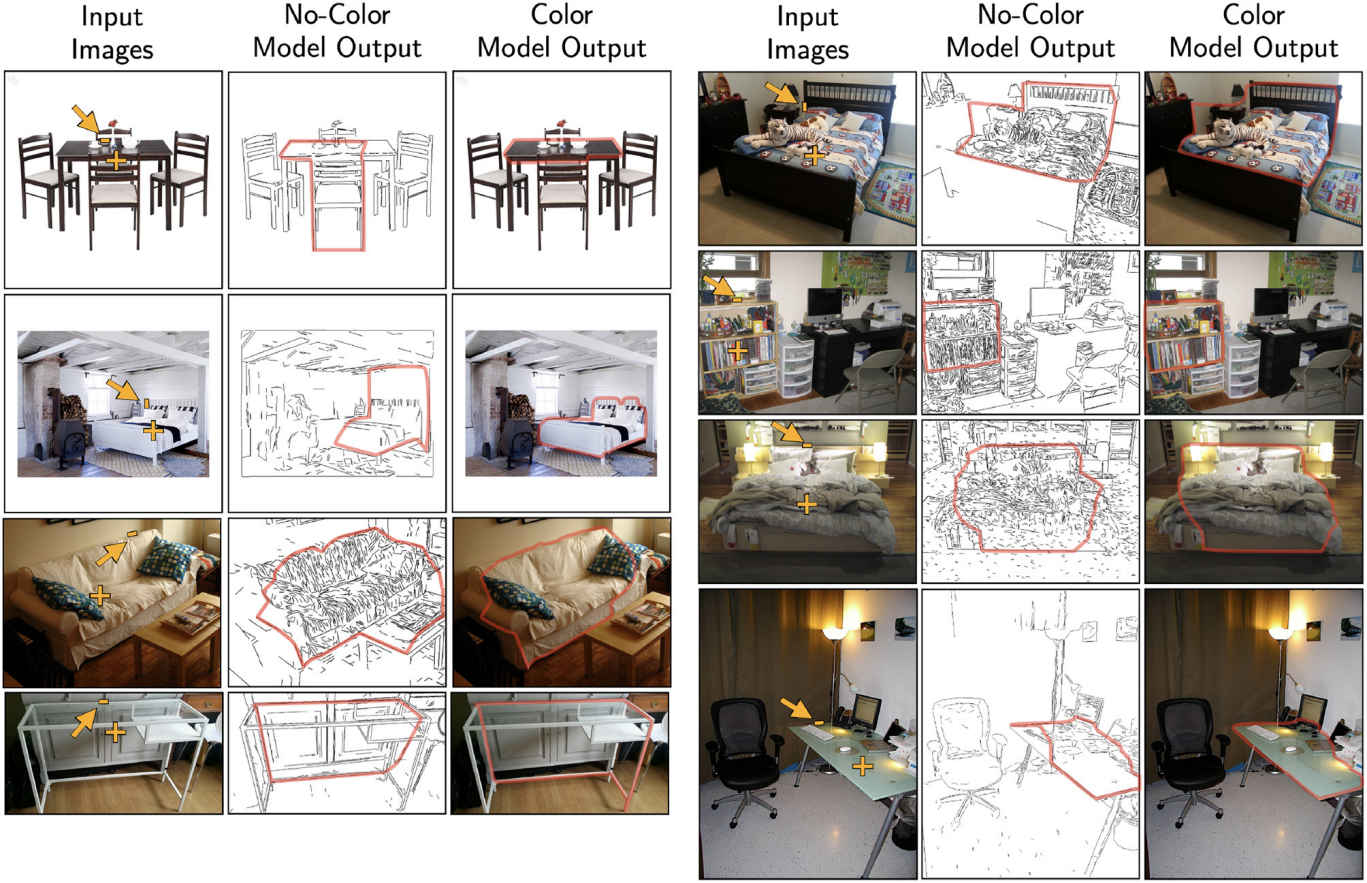
Model performance tested using real images of furniture. The fixation point and starting edge were marked. **Left**: shows the cases where the model produced a different output when color information was used. **Right**: illustrates the stability of model such that adding color produced minimal differences when the no-color model could produce reasonable outputs.

In order to reveal the relationship between contour and color, our present study used synthetic images to increase the difficulty of the task so that subjects would not perform at ceiling. Using synthetic stimuli also allowed us to manipulate contour and color cues independently to control the difficulty across conditions. Since the model could replicate human contour-color interaction using these difficult synthetic stimuli, one could expect that the model should be able to extract boundaries using real world images, which typically are easy for human observers. Without any additional tuning of the model parameters, two versions of the model (no-color and color) were applied to real images of furniture from the Pix3D dataset (Sun et al., 2018). Specifically, we used the coefficients from the jitter 20° random-background condition, which better resembled the amount of noise in real images. Two sets of coefficients were tried: [1,0.4,0,0.8] from distortion *k* = 0.04 and [1,1.2,4,0] from distortion *k* = 0.08. Both sets of coefficients produced similar outputs. Given an input image, an additional pre-processing stage of edge detection was applied (Lee et al., 2014). The no-color model received only the detected edges as input; whereas the color model received additional color information associated to the left and right regions of each edge. Fixation point was placed inside the shape, and a random edge belonging to the target shape was given as the initial edge. Figure 15 shows examples of the model output tested on five different categories of furniture: table, bed, sofa, desk, and bookcase. The preliminary results suggest that the model could be applied to a wide variety of real images. Future studies could test the model generalizability by using real images from different domains.

Another topic for future research is to integrate saliency maps with the model. Because the model used the log-polar representation, there is a requirement for the fixation point to be placed inside the boundary of the target object. Previous literature has suggested that humans use sophisticated attention mechanisms to guide fixation, Schütz et al. (2011), one example being the salience network for bottom-up processing. This network integrates different features such as orientation, color, or motion to create a saliency map which highlights the regions in the image that are most relevant for fixation (for a review, see Uddin, 2016).

Finally, the boundary extraction model could be used as a front-end model for higher order visual processing such as 3-dimensional (3D) object reconstruction. We already showed that if the symmetry correspondence problem is solved, 3D shape recovery can be accomplished (Pizlo et al., 2014). However, solving 3D symmetry correspondence for several objects in a 2D camera image is computationally challenging, if possible at all. Restricting the symmetry correspondence analysis to one object at a time will be likely to produce acceptable solutions.

## Data availability statement

Stimuli used for this study can be found on The Open Science Framework: osf.io/fq5hu, further inquiries can be directed to the corresponding author.

## Ethics statement

The studies involving humans were approved by UCI Institutional Review Board. The studies were conducted in accordance with the local legislation and institutional requirements. The participants provided their written informed consent to participate in this study. Written informed consent was obtained from the individual(s) for the publication of any potentially identifiable images or data included in this article.

## Author contributions

DH and ZP contributed to conception and design of the study and contributed to manuscript preparation. DH collected and analyzed psychophysical data, formulated the model, and performed simulations. All authors contributed to the article and approved the submitted version.

## Footnotes

^1^For example, consider a circle with its center coinciding with the center of the retina. Inscribe a square into the circle. The perimeter of the circle is longer than that of the inscribed square in the Cartesian coordinates representing the retina. This relationship is flipped in the log-polar space. The circle maps into a straight line, while the square maps into a four-cornered curve (see Figure 13 in Kwon et al., 2016). It follows that the straight line representing the circle is shorter than the curve representing the square.

^2^Multiresolution pyramid adjusts the resolution of operations according to the scale of the object on the retinal image The log-polar architecture is scale invariant such that retinal shapes that differ in sizes are processed by the same number of neurons. Thus, the problem of adjusting scale and resolution of processing is solved naturally by a log-polar transformation.

^3^Nevertheless, *d*′ follows a similar pattern: higher jitter leads to systematically lower *d*′.
